# Preparation of Cavities and How to Fill Them

**Published:** 1891-07

**Authors:** D. R. Jennings

**Affiliations:** Cleveland, O.


// THE DENTAL REGISTER.

VOL. XLV.] JULY, 1891. [No. 7.

### COMMUNICATIONS.

### Preparation of Cavities, and How to Fill Them.

BY DR. D. R. JENNINGS, CLEVELAND, O.

Read at a meeting of the Cleveland, Ohio, Dental Society, June 18th, 1891.

The subject that I am to present is one that can hardly be touched but you will say that some one has said something similar. Therfore, if I say something that you have heard, don't accuse me of plagarizing. There is so much to be said on this subject that I am fearful that I can not tell it so that you will understand why I think there are so many failures, and so very few successes. It is always very much easier to preach than to practice and it is particularly so in this case.

The straw that I am about to thrash may not yield any thing very great, but in my observations during thirty years of continued practice, I have seen quite a large amount where the flail was not used quite as much as it should have been.

My subject is the Preparations of Cavities and How to Fill Them. (Seems to me I hear the whisper going around the room, "Chestnuts.") The present time I think is given too much to the prophylactic instead of what we actually have to do. People come to us with teeth decayed and they want them saved and made useful members. They do not come to us to be told that if their grandfathers and grandmothers had lived so-and-so, and if their fathers and mothers had done so-and-so, a visit to us would not have been necessary; they come to have their teeth saved from further decay and trouble, and that is what I shall try to tell my way of doing.

The example I will use to illustrate my ideas will be the posterior proximal surface of a first, lower, left bicuspid.

Now, I don't want you for one moment to think that I am conceited enough to claim there is no way but my way. I have tried long and hard to learn some positive principle by which we can arrive at as high a degree of the certainty of success as in any other business.

It has been said that the average duration of fillings in proximal cavities in bicuspids is less than three years. Now, if that be true I do not think we can boast very much over our skill in saving teeth in that locality, and I don't undertake to dispute it if all the operations made in that locality by those calling themselves "dentists" are to be counted in the making up of the average. I think, perhaps, that locality is the hardest of all to fill successfully.

The cavity quite large—extending below the margins of the gum, covering almost the entire proximal surface, and quite close to the pulp chamber—after the dam is properly adjusted and a wedge placed between the teeth so that it will force the gum and dam below the bottom of the cavity if possible; then with mallet and chisel cut all the tooth-substance away that will be any hindrance to your seeing all the cavity; then use a diamond disk to true the walls of the tooth; then take a sharp excavator and cut all the softened dentine from the cavity; next, use sharp burrs and cut along the walls so as to have some thing of an anchorage, and if the walls are too frail to hold the filling cut two slots across the top of the tooth to act as supporters or anchors to the filling. Then cut the bottom of the cavity perfectly flat so that there will be no sharp edges to chip or break away when the filling is being driven to it. With another instrument cut a shallow groove quite near the pulp-chamber across the cervical wall so that in case of biting where great pressure is brought to bear on the filling, it would not allow it to break from its foundation.

Be sure to have the walls of the cavity flared enough to make a perfect contour of the tooth, and leaving no flattened space produced by the diamond disk. Have them as smooth as possible so that the gold can be packed to them with ease, without fear of fracture. Now that you have your cavity thoroughly prepared, I should use a loop-matrix—one made so that when the screw is tightened it will clasp the neck of the tooth quite closely, and with flare enough to leave the crown sufficiently contoured to finish and retain its proper shape.

Now we are ready to commence the introduction of the filling. I should first lay a mat of Robinson's Fibrous Filling in the bottom of the cavity, packing it nicely and solidly to the bottom, and adding to that No. 40 cohesive gold cut in strips about two-thirds as wide as the cavity with instruments adapted to that kind of cavity—all the time keeping the gold as nearly level as possible, always working from the center to the walls, (for the reason that gold has a tendency to curl or crawl, and if it is driven toward the walls all the time it is forced to stay there). When near the grinding surface I use lower numbers—No. 4 rolled in ropes so that I can use ovoid or oval-shaped points on my pluggers, but be sure to build up full enough to have material in abundance to finish to the natural contour of the tooth.

Then remove your matrix and with a thin burnisher burnish to the wall as well as possible the surplus or prominence of filling.

Next, take one of the thickened-rim disks and polish the cervical part of the filling to a perfect conformity with the neck of tooth. Use finishing-burrs of the right shapes and sizes to cut the grinding surface to the proper shape. Burrs are better than corrundums as they are not as liable to cut the thick enamel and injure the tooth. Next, use disks of various grades of fineness until the filling is in proper shape.

Finish with cuttle-fish-bone paper disks with a little vaseline on it, and you have left a fine, dead finish.

I hope these remarks will call forth a discussion that will be beneficial to us all.

---

Professional ethics is a system of morals formulated out of unwritten laws that by common consent are recognized, at least, in associated life. DR. S. B. BARTHOLOMEW.

